# Lactate in Drainage Fluid to Predict Complications in Robotic Esophagectomies—A Pilot Study in a Matched Cohort

**DOI:** 10.3390/jcm14176190

**Published:** 2025-09-02

**Authors:** Julius Pochhammer, Sarah Kiani, Henning Hobbensiefken, Hilke Hobbensiefken, Benedikt Reichert, Terbish Taivankhuu, Thomas Becker, Jan-Paul Gundlach

**Affiliations:** Department of General, Visceral-, Thoracic-, Transplantation- and Pediatric Surgery, University Hospital Schleswig-Holstein (UKSH), Campus Kiel, Arnold-Heller-Str. 3, 24105 Kiel, Germany

**Keywords:** anastomotic leakage, Ivor Lewis, prediction of complications, lactate, esophageal resection

## Abstract

**Background/Objectives**: Despite advances in minimally invasive procedures, anastomotic leakages (ALs) after esophageal resections mark the most feared complication. Its early detection can lead to quick interventional treatment with improved survival. Nonetheless, early detection remains challenging, and scores are imprecise and complex. **Methods**: In our study we analyzed mediastinal drainage fluid to find parameters suggesting AL even before it became clinically evident and correlated them to routine biomarkers. All patients with AL after robotically assisted esophageal resections were included and matched 1:1 with uneventful controls. Additionally, transhiatal distal esophageal resections operated during this period were included. Drainage fluid was collected on postoperative days (PODs) 1–4 with consecutive blood gas analysis. Test quality was determined by the area under the curve (AUC) of the receiver operating characteristic curve (ROC). **Results**: In total, 40 patients were included, with 17 developing AL. There were no significant differences in gender, age, BMI or oncological treatment. The 30-day morbidity rate was 65.0%. The study was restricted to events in the first 12 days. While lactate value in drainage fluid differed significantly from POD 3 onwards in the two groups, serum CRP remained without significant differences. We developed the LacCRP score (CRP/30 + lactate/2). The AUC on POD 3 was 0.96, with a sensitivity and specificity of 100% and 75%, respectively. An estimator of 1.08 was found in multivariate analysis: one-point increase in the LacCRP score increases AL probability by 8%. **Conclusions**: This study demonstrates that postoperative lactate determinations in drainage fluid can predict AL after esophageal resection, and its combination with serum CRP results in a reliable LacCRP score.

## 1. Introduction

Despite development of multimodal treatment on the one hand and significant postoperative morbidity rates on the other, resection of the esophagus remains the basis in the curative treatment of esophageal cancer [1,2]. After the introduction of robot-assisted minimally invasive esophagectomy (RAMIE), it has become an alternative to the conventional open esophagectomy (OE), entailing less surgical trauma without compromising on the oncological quality [3,4,5]. Notwithstanding continuous surgical innovations, esophagectomy is a very demanding operation with particularly serious intra- and postoperative complications. Although, in recent years a decline in the morbidity rates post esophagectomy was seen, rates of postoperative infectious complications (PICs) and mortality are still up to 40% [6,7]. PICs encompass pulmonary and gastric conduit-related complications comprising perforation, bleeding, impaired circulation, and anastomotic leakages (AL), which is the most feared complication. AL can increase postoperative mortality and morbidity and impair patient’s quality of life. Different factors, which can be patient- and tumor-related, as well as the surgeon’s experience, can influence the healing of the anastomosis [8,9].

AL following esophageal resection has an incidence of 5–30% [10,11], with an associated morbidity and mortality of 4–15% [12,13,14]. Nevertheless, mediastinitis with following septicaemia mark the most significant complications after esophagectomy. Mediastinitis-associated mortality rates of up to 80% have been reported [14]. Hence, timely and appropriate diagnosis and treatment of PICs, especially AL, is crucial [15].

While the consequences of different immune response and blood biomarkers in esophageal cancer have been investigated in relation to tumor progression and cancer-related survival with promising results [16], the clinical utility of systemic inflammatory biomarkers to predict and manage AL remains controversial [17].

An increase in C-reactive protein (CRP) and procalcitonin (PCT) can regularly be measured early after operations [18]. Infection levels usually reach their maximum on the second or third postoperative day (POD) and normalize over the course of the first postoperative week in an uncomplicated course [19]. If a PIC is present, the infection values are mostly higher early after surgery and do not significantly decrease during the postoperative course [20]. Hence, it was obvious to correlate infection values of CRP and PCT with the predictability of PICs. Attempts have been made to correlate the predictability of PICs with CRP and PCT serum levels. Scores like the “Noble and Underwood” (NUn) score were published [21,22,23,24]. As these scores usually have a better negative predictive value, they can be used to enable early hospital discharge after major abdominal or colorectal surgery [25,26].

Biomarkers measured in drainage fluid were shown to be possible predictors of complications. Lactate, pyruvate, and glucose levels may show deviations during the first postoperative days (PODs) due to hampered local perfusion and indicate the development of anastomotic leakage [27]. To our knowledge, the predictive value of lactate was evaluated in serum, but not in drainage fluid so far [28]. The current pilot study seeks to assess the predictive value of lactate measured in the fluid of postoperative drainages, combined with postoperative serum biomarkers, to predict AL. Results are compared to other scores.

## 2. Materials and Methods

This trial is part of a prospective project to develop bioinspired nanocomposites for early detection of complications in gastrointestinal surgery and focuses on postoperative deviations of inflammatory parameters. Included in this project were all patients with newly created anastomoses after resection at the intestinal tract in our tertiary academical center between April 2021 and February 2022 when informed consent was given. A subgroup of these patients was used for this study. Pre- and postoperative blood samples were collected, and postoperative secretions were taken daily from drainage close to the anastomosis and analyzed using various methods. Patient characteristics were collected from the clinical research database of the oncological biobank BMB-CCC of the Medical Faculty of the University of Kiel and from patient files. The study was approved by the local ethics committee of the Medical Faculty, Kiel University (reference no. A110/99), according to the Declaration of Helsinki. Only de-identified data were used for further analysis.

### 2.1. Patient Cohort

In this pilot study, the data of patients after esophageal resections were analyzed. All patients with AL after the Ivor Lewis procedure were included and matched 1:1 with Ivor Lewis procedures without AL. We also included all transhiatal distal esophageal resections in this period, since due to lower case numbers and a small number of ALs, matching was not possible. All procedures were performed in a standardized manner [3,29].

Prior to neoadjuvant therapy, all patients underwent laparoscopy to rule out peritoneal carcinomatosis. In 9 patients, a percutaneous jejunal feeding tube was inserted during this procedure due to dysphagia or weight loss. Twenty-two patients received a feeding tube during RAMIE to support the postoperative phase with high calorie enteral nutrition, while 9 patients (including all transhiatal esophageal resections) did not receive a feeding tube.

Postoperative care consisted of intensive care unit (ICU) supervision and monitoring and collection of daily blood samples. CRP, PCT and albumin values in serum were determined according to clinical standards. Drainage fluid samples were archived in corresponding tubes, and Lactate, pH and electrolyte values were determined in a common device suitable for blood gas analysis (GEM Premier 3500 blood gas analysis system; Werfen, Munich, Germany) immediately after collection. Verification tests do not yet exist for this procedure, and lactate determination from drainage secretions in a standard serum tube is not reliable in our setting. However, repeat measurements at the beginning showed stable values, the values for each day were normally distributed, and the differences between days were also normally distributed for each patient. We therefore assumed internal validity for our cohort.

### 2.2. Endpoints

The primary endpoint was AL, and the secondary endpoint was PICs of other origins, including pulmonary infectious complications, i.e., pneumonia, pleural effusion, and pleural empyema. AL was defined as an endoscopic or radiological proven defect of the anastomosis at the anastomotic site or gastric conduit with connection from intra- and extra-luminal cavities. Pneumonia was diagnosed by the presence of pulmonary infiltrate on chest X-ray together with elevated CRP levels and/or clinical signs. Pleural empyema was diagnosed by means of computed tomography and subsequent thoracentesis.

### 2.3. Statistical Analysis

The dataset was collected in MS Access (Ver. 2024, Redmond, WA, USA), with further use of JMP 17.2 software (SAS Institute Inc., Cary, NC, USA) for statistical analysis. A Student’s *t*-test was conducted for baseline data and the occurrence of endpoints for normally distributed continuous variables, or the Mann–Whitney–Wilcoxon test for non-normally distributed data. Categorical variables were assessed using the Chi-square test or Fisher’s exact test, as appropriate. CRP, PCT, and lactate levels are reported in their original units. Positively skewed data were log-transformed after distribution assessment. Group comparisons (PIC vs. non-PIC) were conducted using unpaired *t*-tests on the transformed data.

Univariate group analysis was performed using binary logistic regression. For multivariable analysis of the primary endpoints, all independent variables—regardless of univariate significance—were included in a backward stepwise multiple regression. The process was repeated until no further variables could be removed without reducing model performance. The optimal CRP cut-off was determined via ROC analysis and Youden’s J statistic. All *p*-values were two-sided, with *p* < 0.05 considered statistically significant.

## 3. Results

A total of 40 patients who had undergone surgery for carcinoma of the esophagus or gastroesophageal junction were included in the study. Of these, 17 developed AL and 23 did not develop AL. There were no significant differences between the two groups in terms of gender, average age, or BMI. In the group with AL, patients with coronary heart disease and chronic obstructive pulmonary disease (COPD) were found more frequently. Previous oncological treatment did not differ between the two groups (Table 1).

In the group with AL, there was a trend towards more Ivor Lewis esophageal resections; all procedures were robotically assisted. No differences were found with regard to anastomosis placement. In the group with AL, however, the duration of surgery was longer, as was the length of stay in the intensive care unit (ICU) and the entire inpatient stay (Table 2). A 30-day morbidity rate of 65.0% was found in the entire cohort. The most clinically relevant role was played by AL, which became clinically conspicuous on a median of day 8. All patients received endoluminal vacuum therapy, which lasted a median of 22 days (Table 3). Two patients required re-operation.

The lactate value was measured on the first four postoperative days in order to determine the predictive potential of AL in drainage secretions. From POD 3 onwards, the values in the two groups differed significantly. The test quality was determined by the area under the curve (AUC) of the ROC and showed moderate test quality. For comparison, CRP was determined in the serum, where the values did not differ significantly and the AUC showed a significantly poorer test quality.

**Figure 1:**
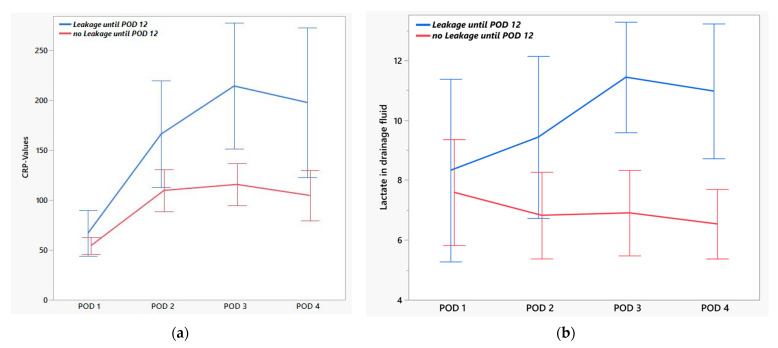
(**a**) Serum C-reactive protein (CRP) and (**b**) lactate detected in drainage fluid early after operation in patients with and without prompt anastomotic leakage. The graphs show mean values; the error bars represent the 95% confidence interval.

**Figure 2:**
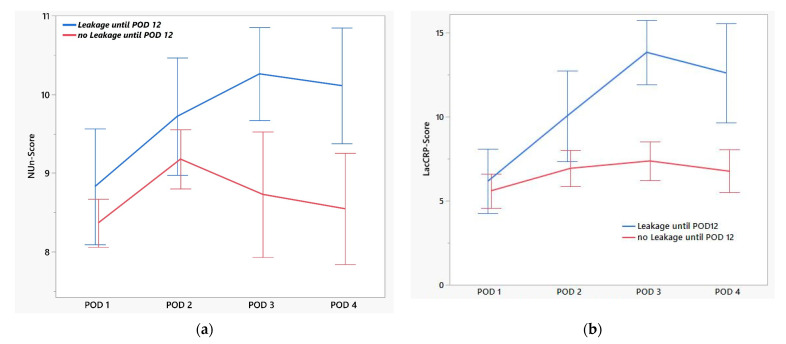
(**a**) Noble und Underwood (NUn) score early after operations in patients with and without prompt anastomotic leakage. The NUn score is calculated for serum C-reactive protein (CRP), white blood cell count (WBC), and albumin [11.3894 + (0.005 × CRP) + (WBC × 0.186) − (0.174 × albumin)]. (**b**) Evolution of LacCRP score early after operations in patients with and without prompt anastomotic leakage. The LacCRP score is calculated for serum C-reactive protein (CRP) and lactate in drainage fluid [CRP/30 + lactate/2]. The graph shows mean values; the error bars represent the 95% confidence interval.

**Figure 3:**
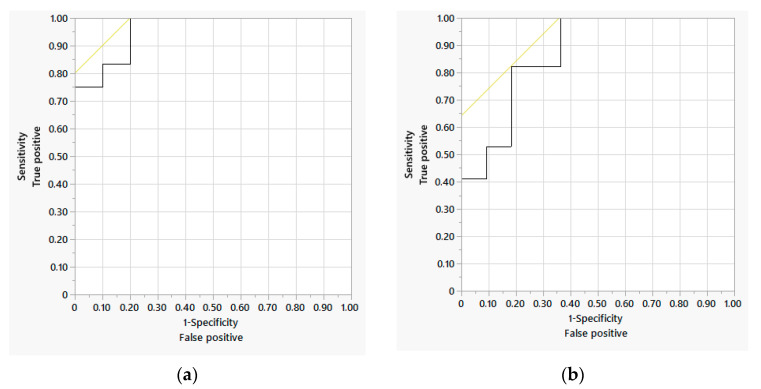
ROC analysis of the predictive quality of the LacCRP score on POD 3 (**a**) and POD 4 (**b**). The AUC is 0.96 and 0.87 on POD3 and POD 4, respectively, with a cutoff of 9.5.

As some ALs occurred late (up to POD 48), we restricted the study to events in the first 12 days. There were significantly different values for drainage lactate from POD 3 and for serum CRP even from POD 2. The AUC improved for both parameters and amounted to 0.85 for lactate and 0.79 for CRP on POD 3 (Table 4, Figure 1a,b). Furthermore, we evaluated the previously described NUn score (calculated from CRP, WBC, and albumin), which showed good test quality in our cohort on POD 3 (AUC 0.89). Since the calculation of the NUn score is complex [11.3894 + (0.005 × CRP) + (WBC × 0.186) − (0.174 × albumine)], we attempted to establish a simpler score. The combination of serum CRP and drainage lactate showed the best results and was easy to calculate [LacCRP score; CRP/30 + lactate/2] (Table 5, Figure 2a,b). The AUC of the ROC for the LacCRP score on POD 3 was 0.96, with a cutoff of 9.5, while the specificity in our cohort was 75%, with a sensitivity of 100% (Figure 3a,b). The multivariate analysis yielded an estimator of 1.08, meaning that each one-point increase in the LacCRP score increases the probability of AL by 8%.

After POD 4, four ALs occurred, on average on POD 22 (15–48). The median LacCRP score was 5.3 (2.4–7.6) and 4.2 (1.5–11.3) on PODs 2 and 3, respectively.

In addition, serum lactate was measured during the postoperative ICU stay and compared with lactate in drainage fluid. The mean values (+SD) were 1.4 + 0.6, 1.2 + 0.6, 1.2 + 0.5, and 1.1 + 0.3 on PODs 1, 2, 3, and 4, respectively. There was no statistically significant correlation; the Pearson correlation coefficients were 0.3, −0.03, −0.11, and −0.47 (the corresponding *p*-values were 0.08, 0.90, 0.71, and 0.21).

## 4. Discussion

This study shows that postoperative lactate determinations in the fluid of mediastinal drains may be a valuable biomarker for the prediction of AL early after esophageal resection. Patients with AL demonstrated a significant increase in lactate levels in their drainage fluid from POD 3 on. The difference persisted until AL became clinically evident, while lactate levels in patients without AL remained stable. There was a clear correlation, particularly for AL occurring in the first 12 days, with a trend towards an increase from POD 2 onwards. To the best of our knowledge, lactate in serum has already been evaluated, but this is the first study to routinely determine lactate in drainage secretions [30].

Our results are in accordance with the existing literature: early postoperative changes in the environment around the anastomosis seem to correlate with the occurrence of AL. Serum markers such as CRP also show a significant increase from POD 3 onwards, but the variation ranges are very wide and the test quality (measured by the AUC of a ROC) is not satisfactory [31].

This demonstrates the need for biomarker-driven strategies in line with previous studies [32]. Although CRP and IL-6 in serum are established and proven indicators of systemic inflammation for the prediction of AL, they have a low test accuracy [31].

Scores such as the NUn score were therefore developed and verified in cohorts. However, their reliability is controversially discussed [31,33]. This score requires a complex calculation and the inclusion of CRP, WBC, and albumin. We therefore combined the lactate determination from drainage secretion with serum CRP: we were able to offer a score with a very good prediction for AL up to day 12 after resection with a simple calculation (lactate/2 + CRP/30) (a sensitivity of 100% and specificity of 75% for a cut off of 9.5).

Local biomarkers have a higher predictive value than serum-based markers as neutrophil-driven local inflammation precedes systemic responses and allows earlier detection already on POD 3 compared to the previously reported POD 5–7 [33]. Compared to conventional serological markers of systemic infection such as CRP and IL-6, measurements from drainage fluid can robustly depict localized early inflammatory changes [34]. These advances enhance risk-tiered postoperative management and provide an advanced methodology designed to overcome limitations in existing surveillance protocols. For example, a cut-off value could be set, above which a gastroscopy or abdominal CT scan would always be performed in order to rule out bias on the part of the surgeon. This could also lead to earlier treatment of AL. If the cut-off value is not exceeded, it could be agreed that certain diagnostic measures would not be necessary.

In this study, we concentrated on AL during the first 12 days. AL also occurred at very late time points (up to POD 48), but an early estimation seems to be infeasible for these late insufficiencies, as a different, yet unexplained, mechanism of development is involved.

For everyday use, ease of use is possible; lactate determination from drainage secretions can be performed using a conventional system for blood gas analysis, which is available in almost every intensive care unit. As described above, we assume internal validity based on the distribution of values. However, we have not yet been able to prove external validity, which represents a relevant limitation of this study.

Even though our study provides valuable findings, it is not without limitations. One major limitation is the small number of cases. Secondly, it is a study that was only conducted at one center, so selection bias is possible. In addition, the selection of matched controls could lead to selection bias, including through the inclusion of transhiatal esophageal resections. A subgroup analysis would be possible in this regard, but our study cohort proved to be too small for these analyses. However, a strength of this study is the complete evaluation of drainage secretion by the same investigators, thus reducing inter-rater correlation. Future multicenter studies with larger samples are required to verify our results and define more accurate threshold values. This requires a validation cohort, as the cut-off value we calculated is optimized for the study cohort under investigation.

## 5. Conclusions

The results of our pilot analysis suggest that the postoperative lactate concentration in the drainage fluid has potential as a relevant biomarker for the early prediction of anastomotic leakage in the first weeks after esophageal surgery. We propose a score based on drainage lactate and serum CRP. This offered the best test quality in our cohort. It is easy to calculate, and lactate measurement is uncomplicated. It is possible that assessment on POD 3 could lead to early diagnosis and intervention but conversely could also favor earlier discharge. Future research should focus on increasing the sample size in a multicenter setting.

## Figures and Tables

**Table 1:** Baseline characteristics of patients undergoing esophageal surgery with or without anastomotic leakage. Data are presented as means ± SDs, medians (min–max), or n (%).

	AL (n = 17)	No AL (n = 23)	*p*-Value
Sex (male)	16 (94.1)	17 (73.9)	0.10
Age (years)	66.1 ± 8.8	65.8 ± 10.2	0.91
BMI (kg/m^2^)	27.5 ± 3.2	28.8 ± 4.7	0.33
Preoperative weight loss (%)	2.4 (0–12.8)	4.2 (0–16)	0.83
ASA-Score 2	4 (23.5)	10 (43.5)	
3	13 (76.5)	10 (43.5)	
4	0	3 (13.0)	0.08
Risk factors			
Smoking	3 (17.7)	3 (13.0)	0.69
Arterial hypertension	12 (70.6)	16 (69.6)	0.94
Coronary heart disease	5 (29.4)	1 (4.4)	0.03
Diabetes mellitus	1 (5.6)	3 (13.0)	0.46
COPD	4 (23.5)	0	0.01
Alcohol misuse	1 (5.9)	1 (4.4)	0.83
Entity			
Adenocarcinoma	13 (38.2)	21 (61.8)	
Squamous cell carcinoma	4 (80.0)	1 (20.0)	0.14
Neoadjuvant Treatment			
Chemotherapy	12 (46.2)	14 (53.9)	
Chemoradiotherapy	2 (40.0)	3 (60.0)	0.79
Adjuvant treatment			
Chemotherapy	1 (25.0)	3 (75.0)	
Radiotherapy	0	1 (100)	0.50

ASA: American Society of Anesthesiologists; BMI: body mass index, COPD: chronic obstructive pulmonary disease.

**Table 2:** Perioperative data.

	AL (n = 17)	No AL (n = 23)	*p*-Value
Procedure			
Abdomino-thoracal esophagectomy (Ivor-Lewis)	16 (50.0)	16 (50.0)	
transhiatal esophagectomy	1 (12.5)	7 (87.5)	0.06
Anastomosis			
End-to-side	14 (45.2)	17 (54.8)	
Side-to-side	1 (20.0)	4 (80.0)	
Side-to-end	0	2 (100.0)	
End-to-end	2 (100.0)	0	0.15
Conversion	1 (100.0)	0	0.24
Length of surgery (min)	350.3 ± 59.6	312.2 ± 56.7	<0.05
Unplanned ICU-treatment	17 (47.2)	19 (52.8)	0.07
Length of ICU stay	3 (1–49)	2 (1–11)	<0.05
Length of hospital stay	39 (14–77)	14 (5–30)	<0.01

ICU: Intensive care unit.

**Table 3:** Postoperative complications of patients. Data are presented as numbers (%) or medians (min–max).

Complications	
30 d-morbidity	26 (65.0)
Anastomotic Leakage	
Day of clinical occurrence	8 (2–48)
Endosponge treatment	17 (100.0)
Duration of endosponge treatment (d)	22 (3–55)
Re-operation	2 (5.0)
Pneumonia	5 (12.5)
Pulmonary artery embolism	2 (5.0)
Pancreatitis	1 (2.5)
Postoperative bleeding	4 (10.0)
Esophagobronchial Fistula	1 (2.5)
Postoperative delirium	2 (5.0)
Surgical Site Infection	3 (7.5)
Superficial	1 (2.5)
Organ–space	2 (5.0)
Anastomotic stenosis	2 (5.0)

**Table 4:** Lactate and CRP, estimated in drain fluid during the early postoperative periods. Data are presented as medians (min–max).

	EAL (n = 13)	No EAL (n = 27)	*p*-Value	AUC
Lactate [mmol/L]				
POD 1	7.7 (2.3–15.0)	5.6 (2.0–15.0)	0.68	0.54
POD 2	8.0 (4.1–15.0)	6.0 (2.2–15.2)	0.09	0.67
POD 3	11.2 (7.3–15.0)	6.3 (1.7–15.0)	<0.01	0.85
POD 4	9.7 (6.3–15.0)	6.3 (2.1–11.5)	<0.01	0.85
CRP [mg/L]				
POD 1	49.4 (24.1–148)	59.5 (9.7–90.5)	0.54	0.56
POD 2	131.0 (67.1–375.0)	109.0 (30.6–227.0)	0.04	0.70
POD 3	223.5 (85.0–389.0)	113.5 (20.5–230.0)	<0.01	0.79
POD 4	143.0 (49.2–414.0)	120.5 (13.2–217.0)	0.03	0.72

EAL: early anastomotic leakages until POD 12; No EAL: no anastomotic leakage; CRP: C-reactive-protein; AUC: area under the curve of a receiver operating characteristic curve.

**Table 5:** NUn and LacCRP Score to predict anastomotic leakage. Scores are calculated for CRP, WBC, albumin, and lactate estimated in drain fluid, respectively. Data are presented as medians (min–max).

	EAL (n = 13)	No EAL (n = 27)	*p*-Value	AUC
NUn-Score				
POD 1	8.6 (7.2–11.2)	8.4 (6.5–9.9)	0.30	0.60
POD 2	9.3 (8.1–11.8)	9.1 (8.1–11.0)	0.22	0.64
POD 3	10.3 (9.0–11.5)	9.1 (6.5–10.1)	<0.01	0.89
POD 4	10.6 (8.7–11.3)	8.5 (7.7–9.1)	0.01	0.91
LacCRP-Score				
POD 1	6.1 (2.3–11.3)	5.4 (2.3–10.1)	0.58	0.55
POD 2	9.4 (4.3–20.0)	7.2 (2.4–11.6)	0.02	0.74
POD 3	14.0 (9.6–18.0)	7.5 (1.5–11.4)	<0.01	0.96
POD 4	11.1 (6.5–18.5)	6.7 (3.1–10.8)	<0.01	0.87

EAL: early anastomotic leakages until POD 12; No EAL: no anastomotic leakage; CRP: C-reactive-protein; AUC: area under the curve of a receiver operating characteristic curve; WBC: white blood cell count.

## Data Availability

The raw data supporting the conclusions of this article will be made available by the authors on request.

## Abbreviations

The following abbreviations are used in this manuscript:

AL	Anastomotic leakages
POD	postoperative days
AUC	area under curve
ROC	receiver operating characteristic curve
CRP	C-reactive protein
RAMIE	robot-assisted minimally invasive esophagectomy
OE	open esophagectomy
PIC	postoperative infectious complications
WBC	white blood cell
PCT	procalcitonin
NUn-score	Noble and Underwood-score
COPD	chronic obstructive pulmonary disease
BMI	body mass index
ICU	intensive care unit
